# Mitofusion-2-mediated alleviation of insulin resistance in rats through reduction in lipid intermediate accumulation in skeletal muscle

**DOI:** 10.1186/1423-0127-20-45

**Published:** 2013-07-01

**Authors:** Xuemei Zhang, Chao Wang, Guangyao Song, Kexin Gan, Dexian Kong, Qian Nie, Luping Ren

**Affiliations:** 1 Hebei Medical University, Shijiazhuang, 050030 Hebei, China; 2 Department of Clinical Medical Research Center, Hebei General Hospital, No348, Heping West Road, Shijiazhuang, Hebei 050071, China; 3 Department of Endocrinology and Metabolism, Hebei General Hospital, No348, Heping West Road, Shijiazhuang, Hebei 050071, China

**Keywords:** Mitofusion2, Insulin resistance, Skeletal musle, Lipid intermediates, CPT1, CD36

## Abstract

**Background:**

Increased lipid accumulation and mitochondrial dysfunction within skeletal muscle have been shown to be strongly associated with insulin resistance. However, the role of mitofusion-2 (MFN2), a key factor in mitochondrial function and energy metabolism, in skeletal muscle lipid intermediate accumulation remains to be elucidated.

**Results:**

A high-fat diet resulted in insulin resistance as well as accumulation of cytosolic lipid intermediates and down-regulation of MFN2 and CPT1 in skeletal muscle in rats, while MFN2 overexpression improved insulin sensitivity and reduced lipid intermediates in muscle, possibly by upregulation of CPT1 expression.

**Conclusions:**

MFN2 overexpression can rescue insulin resistance, possibly by upregulating CPT1 expression leading to reduction in the accumulation of lipid intermediates in skeletal muscle. These observations contribute to the investigations of new diabetes therapies.

## Background

Type 2 diabetes is characterized by insulin resistance (which affects skeletal muscle, liver and other insulin-sensitive tissues) and by defective insulin secretion [1]. Increased adiposity and lipid accumulation within the skeletal muscle and mitochondrial dysfunction have been shown to be strongly associated with insulin resistance [2]. Important studies have appeared in recent years to reinforce the view that it is active lipid intermediates such as long-chain fatty acid CoAs (LCCoAs), diacylglycerols (DAG) and ceramides (CEA) that result in lipid-induced muscle insulin resistance [3,4].

Skeletal muscle is quantitatively the major contributor to whole body insulin-mediated glucose disposal. Fat accumulation in muscle negatively impacts insulin-mediated glucose uptake. The hypothesis that insulin resistance is related to lipid accumulation in muscle dates back to studies reported 15–20 years ago showing that triglycerides accumulate in muscle in rats fed a high-fat diet, which is coincident with insulin resistance. Since then the relevance of muscle lipid accumulation to insulin resistance in humans has been demonstrated, and basic studies have indicated plausible mechanisms whereby lipid accumulation could generate insulin resistance [5].

There may be multiple metabolic causes for increased cytosolic lipid accumulation in muscle in insulin-resistant states. Theoretically, a decrease in fatty acid β-oxidation can lead to lipid accumulation. The acceleration of fatty acid β-oxidation may lessen the potential for insulin resistance [6,7].

CPT1 is the rate-limiting enzyme that controls the transfer of cytosolic LCCoAs into mitochondria for oxidation. Slight increases in CPT1 expression in muscle can direct lipids away from storage and into oxidation, at least in rodents, indicating the importance of this regulatory step in opposing cytosolic lipid accumulation [8]. Mitochondrial dysfunction has been reported in skeletal muscle in type 2 diabetic patients [9-11]. There is evidence that insulin-resistant obese individuals with type 2 diabetes have approximately 30% fewer mitochondria in their skeletal muscle than age-matched healthy controls [12]. It is likely that decreased mitochondrial mass is a defect in type 2 diabetes.

Mitofusin-2 (MFN2) protein is a dynamin-related protein with GTPase activity anchored in the external mitochondrial membrane [13]. MFN2 is abundantly expressed in skeletal muscle [14-16] and its activity is crucial in the maintenance of mitochondrial tubules in cultured muscle cells [17]. Overexpression of MFN2 in HeLa cells causes perinuclear aggregation of mitochondria, a marked enhancement of mitochondrial membrane potential and increased glucose oxidation [17]. Detection of mitochondrial function includes measures of fatty acid oxidation and oxidative phosphorylation. Indicators of fatty acid oxidation potential include measurements of enzymes associated with fatty acid transport protein into the cell (fatty acid transport protein (CD36) [18], fatty acid transport into the mitochondria (carnitine palmitoyltransferase I [CPT1] [19], and fatty acid β-oxidation (β-hydroxyacyl-CoA dehydrogenase [HADH] [20].

Previous studies confirmed that high-fat diets induced insulin resistance and MFN2 down-regulation in rat muscle [21] (David Sebastián, et al. 2012). However, the roles of MFN2, a key factor for mitochondria function and energy metabolism, in skeletal muscle lipid intermediate accumulation remain to be elucidated.

In this study, we established a model of insulin resistance in rats by provision of a high-fat diet. Furthermore, an MFN2-expressing adenovirus was used to investigate the mechanism by which MFN2 relieves skeletal muscle lipid intermediate accumulation and ameliorates insulin resistance.

## Methods

### Animals

The animal experiments were conducted according to protocols reviewed and approved by the Animal Experimental Ethics Committee of Hebei General Hospital, Shijiazhuang, China (approval ID: HBGH-2010027). Four-week-old male Sprague Dawley (SD) rats (70 ± 10 g) were housed individually at 22°C, in 12 h/12 h light/dark conditions and 50% relative humidity. SD rats were divided randomly into two groups of 36: normal diet (8 N) group (n = 12), high-fat diet (8 F) group (n = 24). The 8 N group was fed with a standard carbohydrate diet (10.3% fat, 24.2% protein, and 65.5% carbohydrate), while the 8 F group was fed with a high-fat diet (59.8% fat, 20.1% protein, and 20.1% carbohydrate). After 8 weeks of feeding, insulin resistance in each group was evaluated by glucose infusion rate (GIR) using the hyperinsulinemic euglycemic clamp technique in the conscious state. Six randomly selected rats in each group were then sacrificed by exsanguination. Fasting blood glucose (BG) levels were measured using an Accu-chek Active Meter (ACCU-CHEK^®^ Active; Roche,Basel,Switzerland), Fasting serum insulin levels were determined with a rat insulin ELISA kit (Crystal Chem. Inc.). Skeletal muscle tissue was rapidly removed, freeze-clamped in liquid nitrogen and stored at -80°C. The high-fat diet (8 F) group was divided randomly into three groups: high-fat diet control (control) group, empty adenovirus (Ad) group, and MFN2 overexpression (Ad-MFN2) group. All rats were then infected with PBS as a control, empty adenovirus or the MFN2 overexpressing adenovirus (10^9^ v.p./kg body weight) once a week for 3 weeks. The MFN2 overexpressing adenovirus (Ad-MFN2) and the empty control adenovirus (Ad) were obtained from Dr Rui Zhang, Hebei Medical University [22]. Except for the periods of pretest overnight fasting and the immediate postoperative period, animals had free access to water and chow. Hyperinsulinemic clamp studies were repeated to confirm the amelioration of insulin resistance. Overnight-fasted rats were anesthetized with sodium pentobarbital (3% Pelltobarbitalum Natricum, 60 mg/kg, intraperitoneally) and blood samples were obtained from the abdominal aorta. Skeletal muscle tissue was immediately removed, freeze-clamped in liquid nitrogen and stored at -80°C.

### Hyperinsulinemic euglycemic clamp

Insulin sensitivity can be measured using a variety of techniques that are commonly employed in diabetes research and care. Of these, the hyperinsulinemic euglycemic clamp is the gold-standard method. Hyperinsulinemic clamp studies were performed as previously described [23]. Rats were placed under general anesthesia (3% Pelltobarbitalum Natricum, 60 mg/kg, intraperitoneally) and catheters were inserted into the right jugular vein and the carotid arteries of rats and exteriorized from the back of the neck subcutaneously. At the end, the catheters were flushed with isotonic saline containing heparin (50 units/ml). Rats were allowed a minimum of 3 days for full recovery and only those that had lost less than 5% of their preoperative weights were used in experiments. Euglycemic-hyperinsulinemic clamps were performed on fasted, awake, and unrestrained animals. Insulin (4 mU/kg/min) was infused through the jugular vein catheter from 0 to 90 min. Glucose concentrations were clamped at euglycemic levels by a variable rate infusion of 30% glucose. BG levels were monitored with a glucometer (ACCU-CHEK^®^ Active; Roche), and GIR were adjusted every 5–10 min as required. A stable GIR was obtained within approximately 60 minutes of the insulin infusion and maintained thereafter. At steady state, mean GIR was normalized to body weight.

### Electron microscopy

Skeletal muscle samples to be used for electron microscopy were cut into small pieces (1 × 1 × 2 mm) and fixed in 2.5% glutaraldehyde, post-fixed in 1% osmium tetroxide, dehydrated, and embedded in Epon in the longitudinal orientation. After an initial low-power screening of semithin (300 nm) sections stained with toluidine blue to optimize the plane of sectioning, ultrathin (60 nm) longitudinal sections were cut for each sample. The sections were mounted on copper grids and stained with lead citrate and uranyl acetate. For each biopsy, at least 10 longitudinal sections were examined by transmission electron microscopy (TEM) (H-7500, Japanese Hitachi Ltd) at an accelerating voltage of 80 kV. A minimum of 10 micrographs were taken at 15,000 × magnification.

### Determination of skeletal muscle triglycerides, long-chain fatty acid CoAs, diacylglycerol and ceramides

Triacylglycerols were extracted with a 2:1 chloroform-methanol solution and quantified with an enzymatic assay kit (Wako Pure Chemical Industries) as previously described [6]. Extraction of long-chain fatty acid CoAs, diacylglycerol and ceramides via HPLC was performed as previously described [24,25].

### Real-time RT-PCR

Total RNA was extracted from frozen skeletal muscle using a standard TRIzol (Invitrogen, USA) RNA isolation method. Reverse transcription of RNA was carried out according to the instructions of the Easy Script First-Strand cDNA Synthesis Super Mix kit (Trans Gen Biotech, CA). Specific primers designed for amplification of MFN2, CPT1, CD36 and β-actin were verified by NCBI Blast; primers sequences are shown in Table 1. Real-time PCR was performed on an ABI PRISM 7300 PCR System (Applied Bio systems, USA) using SYBR Green I GoTaq^®^ qPCR Master Mix (Promega, USA). PCRs were carried out in a total of 25 μl as follows: one cycle at 95°C for 5 min, followed by 40 cycles of 95°C for 15 s, 58°C for 20 s and 72°C for 30 s. The gene expression from each sample was analysed in duplicates and normalized against β-actin. The results are expressed as relative gene expression using the ΔCt method.

**Table 1:** Primer sequences for quantitative PCR

**Gene**	**Forward primer (5'-3')**	**Reverse primer (5'-3')**
β-actin	CGGTCAGGTCATCACTATCG	GAAGGAAGGCTGGAAGAGAG
MFN2	AGCGTCCTCTCCCTCTGACA	TTCCACACCACTCCTCCGAC
CPT1	CCAGGCAAAGAGACAGACTTG	GCCAAACC TTAGAGAAGCGA
CD36	AATGAGACTGGGACCATCG	CTCCAACACCAAGTAAGACCAT

### Western blot analysis

Frozen skeletal muscle was prepared with lysis buffer (1% Triton X-100, 150 mM NaCl, 10 mM Tris–HCl (pH 7.4); 1 mM EDTA, 1 mM EGTA (pH 8.0); 0.2 mM Na_3_VO_4_, 0.2 mM phenylmethylsulfonyl fluoride, and 0.5% NP-40). Equal amounts of proteins were separated by 10% SDS-PAGE, and electrotransferred to PVDF membranes (Millipore, USA), and then were blocked with 5% BSA for 2 h at room temperature. Membranes were incubated with appropriately diluted rabbit anti-rat primary antibodies for detection of MFN2 (Bioworld Technology, Inc.), CPT1 (Santa Cruz), CD36 (Santa Cruz) or mouse anti-rat β-actin (Cell Signaling Technology) overnight at 4°C. Membranes were then incubated with the relevant secondary antibodies (MFN2, CD36, CPT1: anti-rabbit IgG; β-actin: anti-mouse IgG; all from Santa Cruz) for 2 h at 20°C. Proteins were detected with the enhanced chemiluminescence (ECL) detection system. β-actin was served as an internal control protein.

### Statistical analysis

All experiments were repeated at least three times. Values are represented as means ± SD. All statistical analyses were performed with the SPSS statistical package (SPSS 16.0 software). Statistical significance was assessed by ANOVA (post-hoc used Student-Newman-Keuls test) and unpaired Student’s *t*-tests (*P*-values < 0.05 were considered significant).

## Results

### Evaluation of insulin sensitivity in high-fat diet treated rats

**Figure 1:**
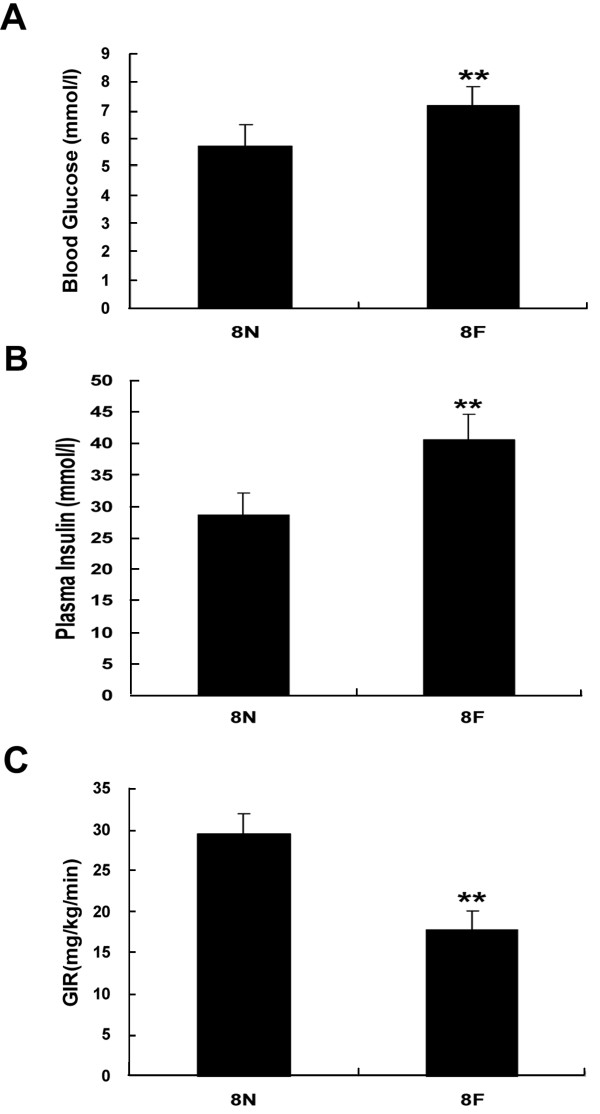
**High-fat diets resulted in insulin resistance in rats.** Rats were fed with high-fat diets or normal diets for 8 weeks, when the levels of blood glucose **(A)** and plasma insulin **(B)** were measured. The GIR, assayed by hyperinsulinemic euglycemic clamping, was used to assess insulin sensitivity in rats **(C)**. **P < 0.01, compared with normal diets (8 N), n = 6.

Fasting plasma glucose levels, serum insulin levels and glucose infusion rates (GIR) in rats are presented as Figure 1. Compared with rats fed a normal diet, high-fat diet rats exhibited obviously impaired insulin sensitivity, indicated by increased levels of fasting plasma glucose (7.2 ± 0.6 mmol/l vs. 5.7 ± 0.7, *P* < 0.01 mmol/l) and fasting serum insulin (40.6 ± 3.9 mU/l vs. 28.7 ± 3.3 mU/l, *P* < 0.01). Compared with control rats, GIR decreased markedly in high-fat diet rats (17.7 ± 2.3 mg/kg/min vs. 29.4 ± 2.4 mg/kg/min, *P* < 0.01).

### High-fat diet increased the accumulation of lipid intermediates in skeletal muscle, and downregulated the expression of MFN2 and CPT1

**Figure 2:**
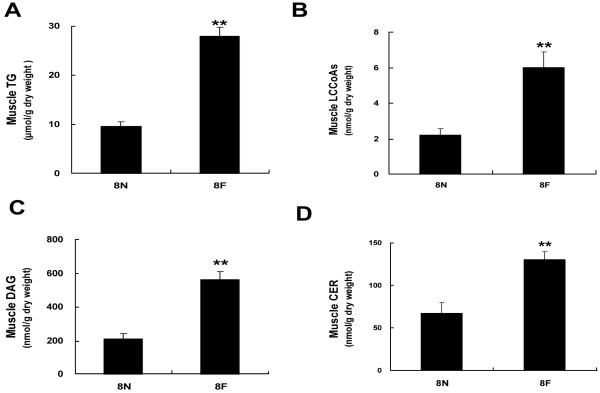
**High-fat diets resulted in lipid accumulation in skeletal muscle.** Rats were fed with high-fat diet or normal diet for 8 weeks, when the levels of TG **(A)**, LCCoAs **(B)**, DAG **(C)**, CER **(D)** were measured. **P <0.01, compared with normal diet (8 N), n = 6.

**Figure 3:**
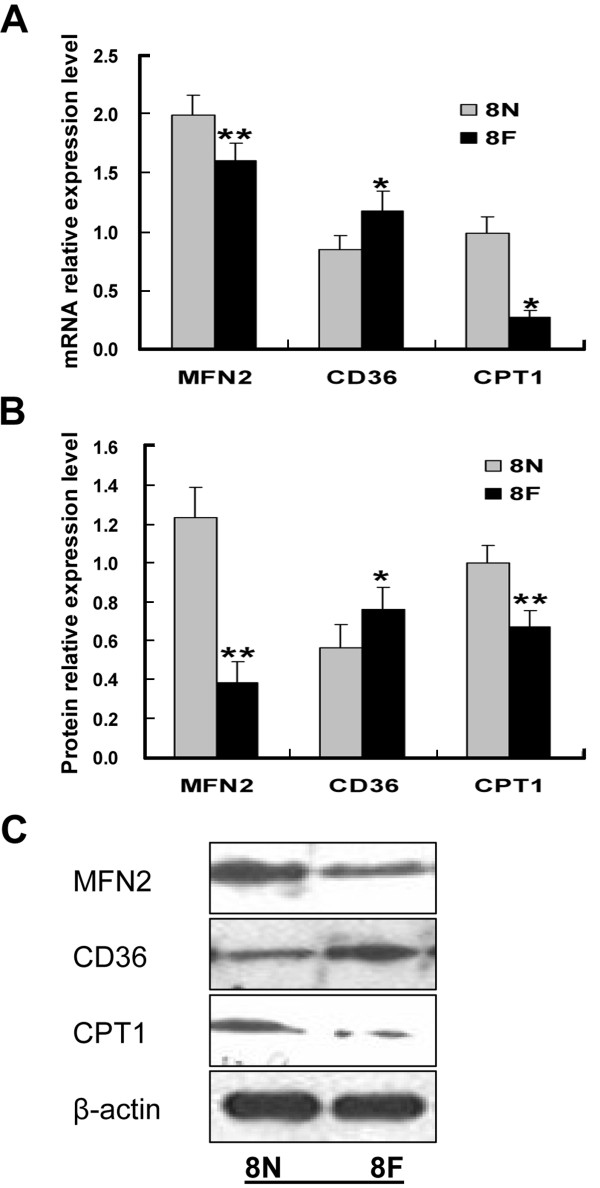
**High-fat diets inhibited MFN2 and CPT1 in rat skeletal muscle.** Rats were fed with high-fat diet (8 F) or normal diet (8 N) for 8 weeks, when the expression levels of MFN2, CPT1 and CD36 were assayed by quantitative RT-PCR **(A)** and Western blot **(B**, **C)**. *P < 0.05, **P < 0.01, compared with normal diets (8 N), n = 6.

As shown in Figure 2, compared with control rats, the levels of lipid intermediates in skeletal muscle of high-fat diet rats were significantly increased (LCCoAs 6.0 ± 0.8 nmol/g vs. 2.2 ± 0.3 nmol/g, DAG 563.0 ± 48.3 nmol/g vs. 211.5 ± 31 nmol/g, CEA 130.3 ± 9.3 nmol/g vs. 67.2 ± 12.2, *P* < 0.01 nmol/g). We also observed a high-fat diet-induced decrease in the expression of MFN2 at both the mRNA and protein levels (Figure 3). The expression of CPT1 was concomitantly decreased significantly (*P* < 0.01) (Figure 3), while the expression of CD36 was increased markedly (*P* < 0.01) (Figure 3).

### Changes in mitochondrial morphology following MFN2 overexpression

**Figure 4:**
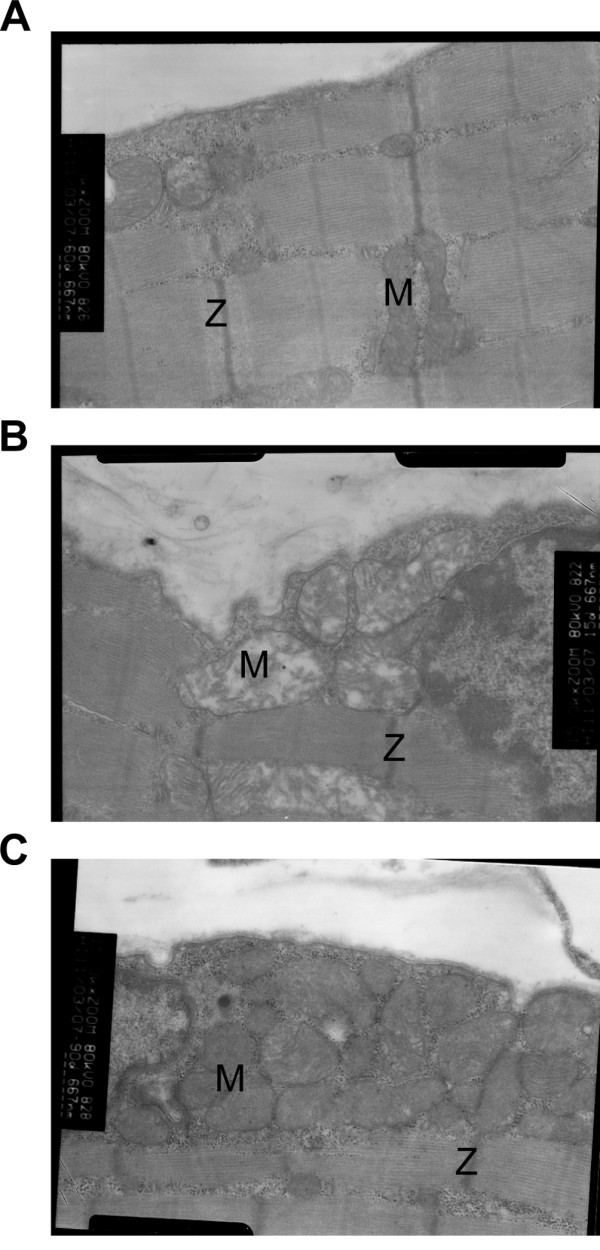
**Representative transmission electron micrographs (original magnification: ×15,000) of skeletal muscle from a normal diet rat (A), a high-fat diet rat (B), and MFN2 overexpressing adenovirus infected (10**^**9 **^**v.p./kg body weight) rat (C).** Several mitochondria are labeled with the letter M. Z-lines are labeled with the letter Z.

The effect of MFN2 on the mitochondrial morphology in skeletal muscle was investigated by electron microscopy. This method allows accurate assessment of ultrastructural morphology as well as the number of mitochondria and cristae. As shown in the representative photographs in Figure 4, mitochondria from muscle in the MFN2 overexpression group exhibited a more clearly defined internal membrane structure, including wider cristae, compared with those observed in the high-fat diet group.

### Overexpression of MFN2 ameliorated insulin resistance induced by a high-fat diet in rats

**Figure 5:**
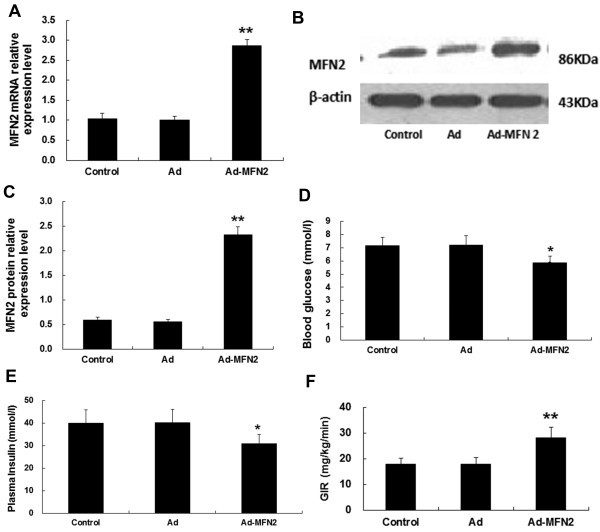
**MFN2 overexpression improved insulin sensitivity in rats.** Rats were fed with high-fat diets for 8 weeks, and then were infected with Ad-Mfn2 (10^9^ v.p./kg body weight) or empty Ad adenoviruses or PBS control for 3 weeks. MFN2 overexpression in skeletal muscle of rats was confirmed by quantitative RT-PCR **(A)** and Western blot analyses **(B**, **C)**. The levels of blood glucose **(D)**, plasma insulin **(E)** and insulin sensitivity (**F**) were assayed. *P < 0.05, **P < 0.01, compared with Ad, n = 6.

n order to investigate the effect of MFN2 on insulin sensitivity, rats were fed a high-fat diet for 8 weeks and then infected with Ad-MFN2 or empty Ad adenoviruses or PBS control for 3 weeks. MFN2 expression in rat muscle was increased dramatically by Ad-MFN2 infection (Figure 5A,B and C). At the same time, fasting BG (Control: 7.18 ± 0.6 mmol/l; Ad: 7.2 ± 0.7 mmol/l; Ad-*Mfn2*: 5.9 ± 0.7 mmol/l, Figure 5D) and plasma insulin levels decreased (Control: 40.0 ± 5.9 mU/l; Ad: 40.1 ± 5.9 mU/l; Ad-*Mfn2*: 30.9 ± 3.7 mU/l, Figure 5E) while GIR increased (Control: 17.74 ± 2.34 mg/kg/min; Ad: 17.91 ± 2.56 mg/kg/min; Ad-*Mfn2*: 28.16 ± 4.71 mg/kg/min, Figure 5F) markedly with Ad-Mfn2 infection. The results indicated that MFN2 overexpression neutralized the effects of a high-fat diet on insulin sensitivity.

### Overexpression of MFN2 improved accumulation of muscle lipid intermediates in high-fat diet rats

**Figure 6:**
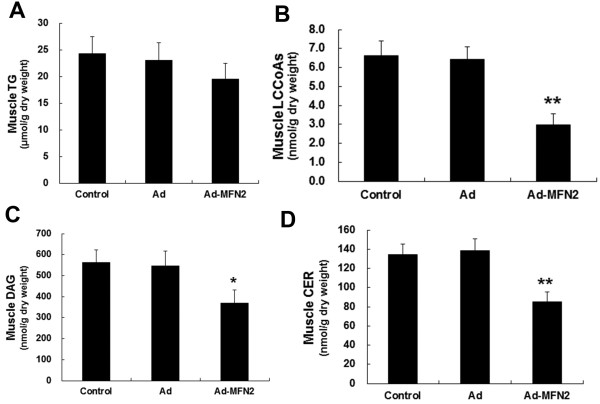
**MFN2 overexpression lowered lipid intermediate accumulation in rat skeletal muscle, but had no significant effect on muscle TG accumulation.** Rats were fed with high-fat diets for 8 weeks, and then were infected with Ad-Mfn2 (10^9^ v.p./kg body weight) or empty Ad adenoviruses or PBS control for 3 weeks. The levels of TG **(A)**, LCCoAs **(B)**, DAG **(C)** and CER **(D)** were measured. *P < 0.05, **P <0.01, compared with Ad, n = 6.

Changes in lipid intermediate accumulation in rat muscle overexpressing MFN2 were detected by HPLC assays. Although there were no significant changes in the accumulation of TG (Figure 6A), the accumulation of lipid intermediates, such as LCCoAs, DAG and CER was markedly reduced (Figure 6B, C and D). These changes were concomitant with improved insulin sensitivity.

### Overexpression of MFN2 increased CPT1 expression

**Figure 7:**
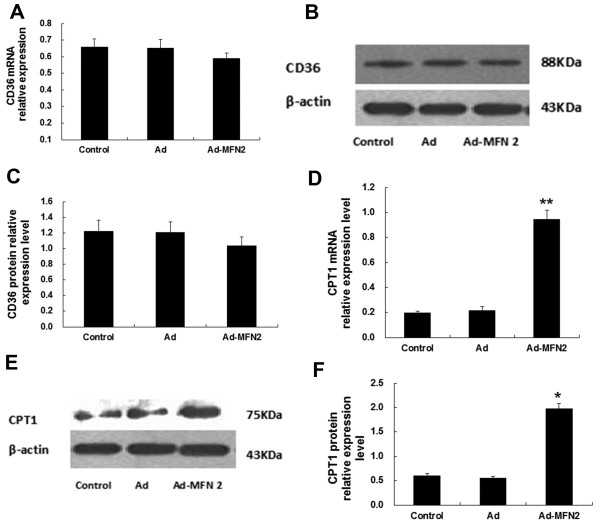
**MFN2 overexpression increased CPT1 mRNA and protein expression in rat skeletal muscle, but had no significant effects on muscle CD36 expression.** Rats were fed with high-fat diets for 8 weeks, and then were infected with Ad-MFN2 (10^9^ v.p./kg body weight) or empty Ad adenoviruses or PBS control for 3 weeks. The expression levels of CD36 **(A**, **B**, **C)** and CPT1 **(D**, **E**, **F)** were measured by Real-time PCR and Western blot. *P < 0.05, **P < 0.01, compared with Ad, n = 6.

As shown in Figure 7, CPT1 was upregulated markedly at both the mRNA and protein levels (Figure 7D, E and F), while the expression of CD36 was not significantly affected (Figure 7A, B and C). The results suggested that MFN2 improvement of insulin sensitivity may be correlated with decreased lipid accumulation.

## Discussion

Our study suggested that MFN2 expression and insulin sensitivity were inhibited by a high-fat diet, with concomitant accumulation of lipid intermediates in the muscle. This is in agreement with previously reported findings [21]. Furthermore, we demonstrated that overexpression of MFN2 recovered insulin sensitivity and reduced lipid accumulation, which may be associated with CPT1 upregulation in skeletal muscle.

Over-consumption of fat is a significant contributor to the development of obesity and insulin resistance. High-fat-diets enriched with lard were widely used to induce an obese animal model of insulin resistance [26]. In this study, lipid accumulation in skeletal muscle was observed in rats fed a high-fat diet, while MFN2 and CPT1 expression was downregulated dramatically and CD36 expression was increased. This may suggest that the lower expression of MFN2 and CPT1 is a consequence of mitochondrial dysfunction, while the higher expression of CD36 is compensational for increased blood plasma free fatty acid levels induced by a high-fat diet. When the levels of fatty acids transported into the cytosol exceed the capacity for mitochondrial oxidation, lipid or lipid intermediates are deposited in skeletal muscle.

Normally, the binding of insulin to insulin receptors stimulates autophosphorylation of tyrosine residues and subsequent activation of a receptor tyrosine kinase. This tyrosine kinase phosphorylates multiple intracellular substrates, including insulin receptor substrate (IRS) 1 and 2, which play significant roles in the insulin response. This is mediated by IRS activation of phosphatidylinositol 3-kinase (PI 3-kinase), a critical player in insulin signaling particularly with regard to glucose homeostasis. Phosphatidylinositol 3-kinase facilitates the translocation of the insulin-responsive glucose transporter (GLUT4) to the plasma membrane through a mechanism that is thought to be mediated by phosphorylation [27]. Recent studies confirm that lipid intermediates such as long-chain fatty acid CoA species (LCCoAs), diacylglycerol (DAG), and ceramides (CEA) disrupt one or more of the early steps in insulin signal transduction. These compounds have been implicated in the activation of isoforms of PKC, leading to impairment of phosphatidylinositol 3-kinase (PI3K) activity and inhibition of the activation of PKB (Akt/PKB) as well as decreased insulin-induced glucose transportation and glycogen synthesis [28].

Mitochondria are the primary cellular sites for fatty acid oxidation and utilization. Mitochondrial dysfunction plays an important role in the development of insulin resistance in skeletal muscle [29]. MFN2 is a proliferation-inhibiting gene encoding a mitochondrial fusion protein that participates in the maintenance of mitochondrial morphology and regulates mitochondrial metabolism and intracellular signaling [16,30]. Although the central role of MFN2 in mitochondrial metabolism is well-established [29], its roles in insulin resistance in skeletal muscle and muscle lipid intermediate accumulation remain uncertain.

Mitochondrial β-oxidation represents a crucial process in energy metabolism and is tightly regulated by interactions between the key enzymes carnitine palmitoyltransferase 1 (CPT1) and acetyl-CoA carboxylase (ACC) via the intermediate malonyl-CoA. CPT1 is an integral membrane protein that associates with the mitochondrial outer membrane through transmembrane regions in the peptide chain. Both MFN2 and CPT1 are regulated by peroxisome proliferators-activated receptor coactivator-1 (PGC1). CD36 is a multifunctional membrane-associated glycoprotein with a molecular weight of 88 kDa. It plays an important role in the uptake of long-chain fatty acids (LCFAs) in skeletal muscle [31]. Superficially, there are no direct association between CPT1 and CD36; however, more recent information suggests that CPT1 may not act alone in the regulation of fatty acyl-CoA entry into the mitochondria. Recent studies have demonstrated that the fatty acid translocase FAT/CD36 is present on the mitochondrial membrane of both rodent and human skeletal muscle, where it plays an important role in regulating fatty acid oxidation [32]. Above all, the elucidation of the highly complex relationships among MFN2, CPT1 and CD36 are of critical significance.

In the current study, our data demonstrate that overexpression of MFN2 significantly restored insulin sensitivity and reduced the levels of BG and plasma insulin in rats, suggesting MFN2 as a potential therapeutic target in insulin resistance. It can be speculated that the mechanism underlying the improvement in insulin resistance associated with MFN2 overexpression is mediated by improved mitochondrial function. Consequently, with the upregulation of CPT1, the redundant lipid intermediates in the cytosolic were transported into the mitochondria for β-oxidation, thus relieving the damage to insulin signaling resulting from the accumulated lipid intermediates.

## Conclusion

In conclusion, our data suggest that MFN2 ameliorates insulin resistance induced by a high-fat diet through upregulation of CPT1, which relieves lipid intermediates accumulation in skeletal muscle. Therefore MFN2 is implicated as a potential target for the treatment of insulin resistance and metabolic syndromes.

## Competing interests

The authors declare that there is no conflict of interest that could be perceived as prejudicing the impartiality of the research reported.

## Authors’ contributions

GYS, CW and XMZ conceived and designed the experiments; XMZ, CW, KXG, DXK and QN performed the experiments; XMZ, CW and GYS analyzed the data; XMZ wrote the paper. All authors read and approved the final manuscript.
